# Supplementary material for: Understanding the impact of mobility on COVID-19 spread: A hybrid gravity-metapopulation model of COVID-19
Source: PLoS Comput Biol. 2023 May 12;19(5):e1011123. doi: 10.1371/journal.pcbi.1011123 (PMC10208486; doi:10.1371/journal.pcbi.1011123)
Supplement: S1 Text — This document contains more details of the methods and results. (PDF) [file pcbi.1011123.s001.pdf]

Supplementary material

A Weekly reported cases of COVID-19

We present the weekly reported cases of COVID-19 in the thirteen local health areas of Fraser Health, BC, Canada, from July 2020 to January 2021, inclusive. The data was extracted from a line list generated by BCCDC Public Health Reporting Data Warehouse (PHRDW), based on symptom onset date or reported date where symptoms onset date is not available.

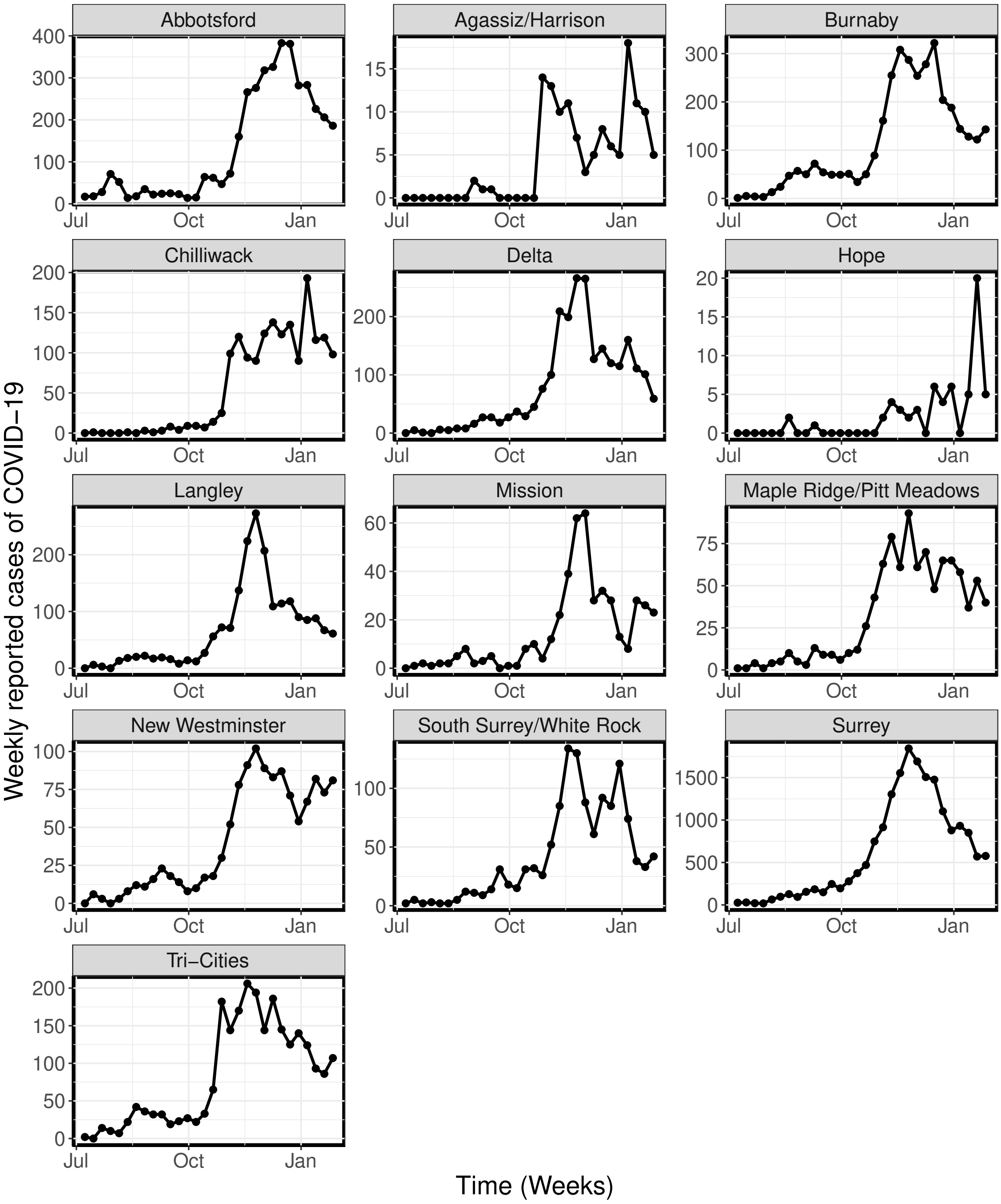

Figure A: ***Reported cases of COVID-19.*** *Weekly reported cases of COVID-19 for the 13 local health areas (LHA) of Fraser Health, British Columbia (BC), Canada, for the period from July 2020 to January 2021.*

B Weekly mobility matrices

We present the weekly mobility matrices constructed from Telus mobility data for the period from July 2021 to January 2022.

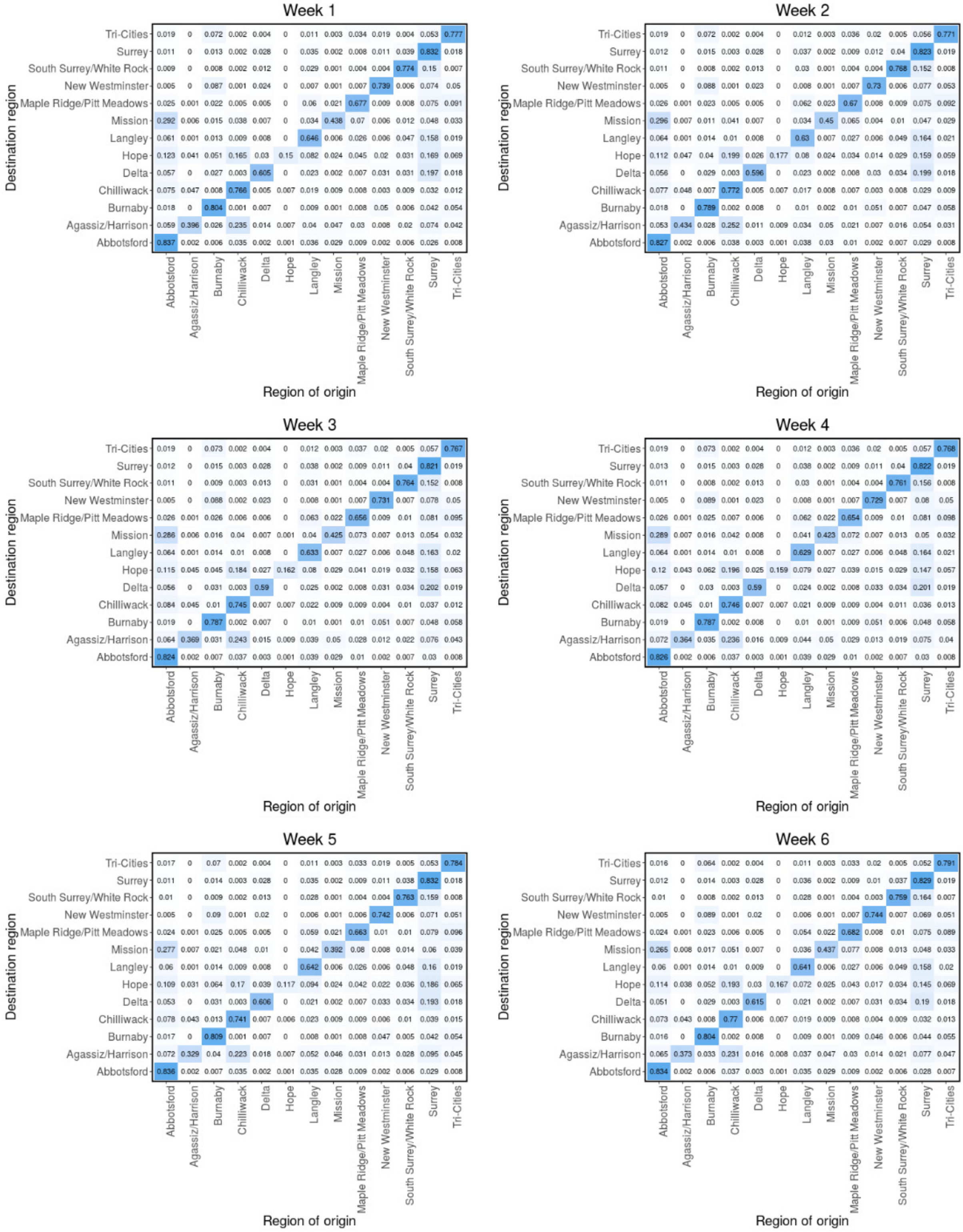

Figure B: ***Mobility matrices****. Probability matrix (π) computed from the Telus mobility data for week 1 - 6 (July 1, 2020 - Aug 12, 2020). π_{ji} is the probability that an individual who migrated from one of the 13 LHAs to region j, originated from region i.*

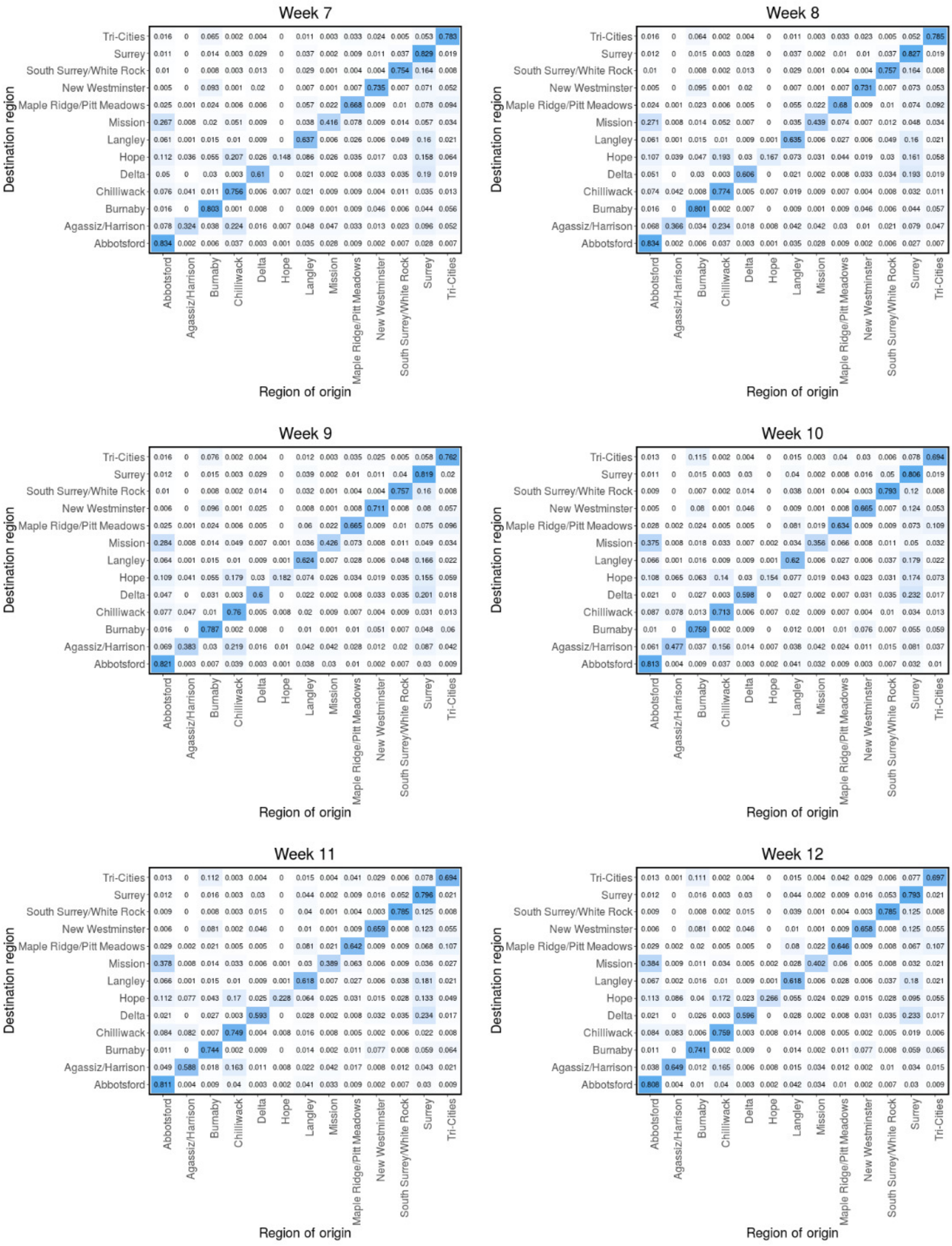

Figure C: ***Mobility matrices****. Probability matrix (π) computed from the Telus mobility data for week 1 - 6 (Aug 13, 2020 - Sept 23, 2020). π_{ji} is the probability that an individual who migrated from one of the 13 LHAs to region j, originated from region i.*

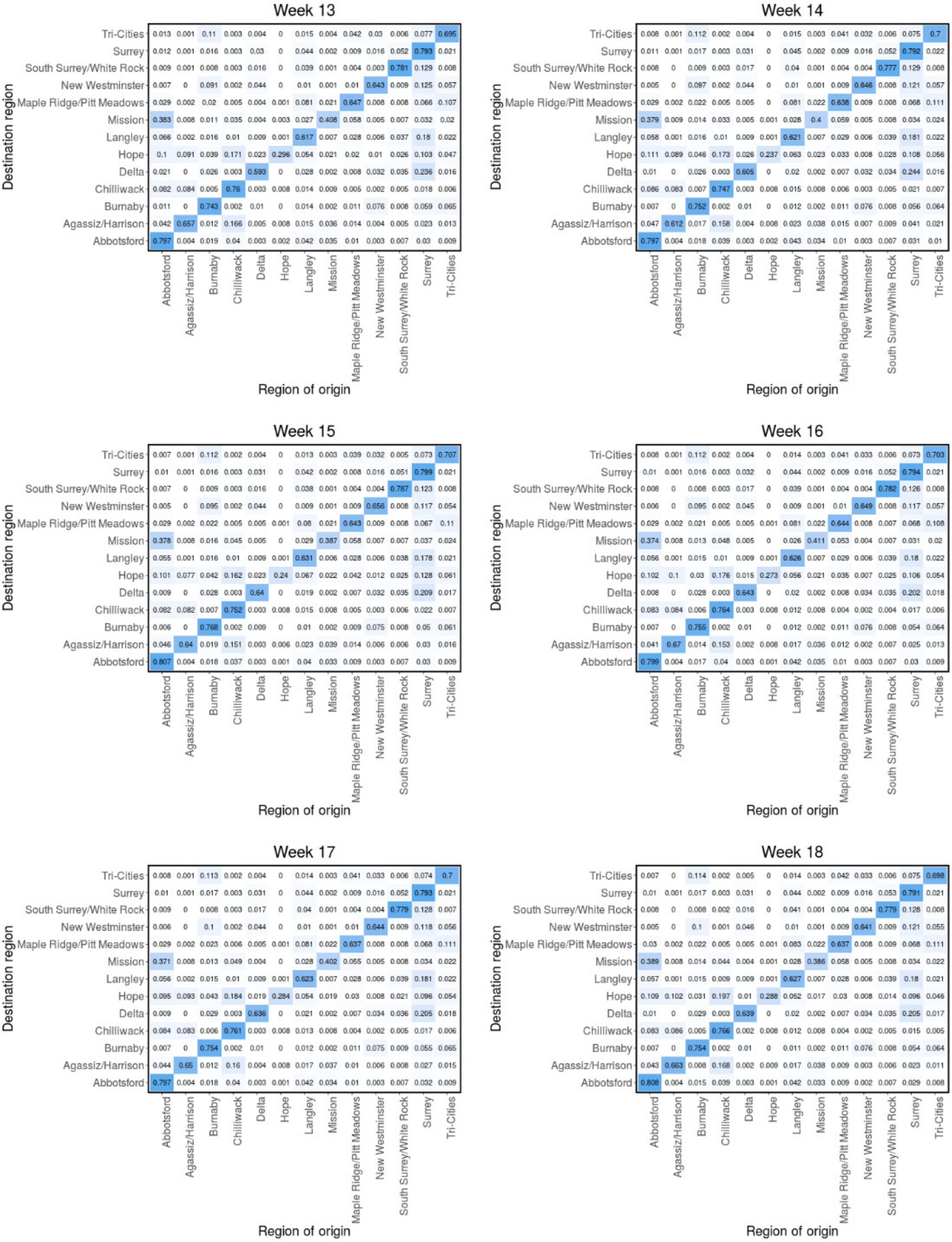

Figure D: ***Mobility matrices****. Probability matrix (π) computed from the Telus mobility data for week 1 - 6 (Sept 24, 2020 - Nov 4, 2020).* π_{ji} *is the probability that an individual who migrated from one of the 13 LHAs to region* j*, originated from region* i*.*

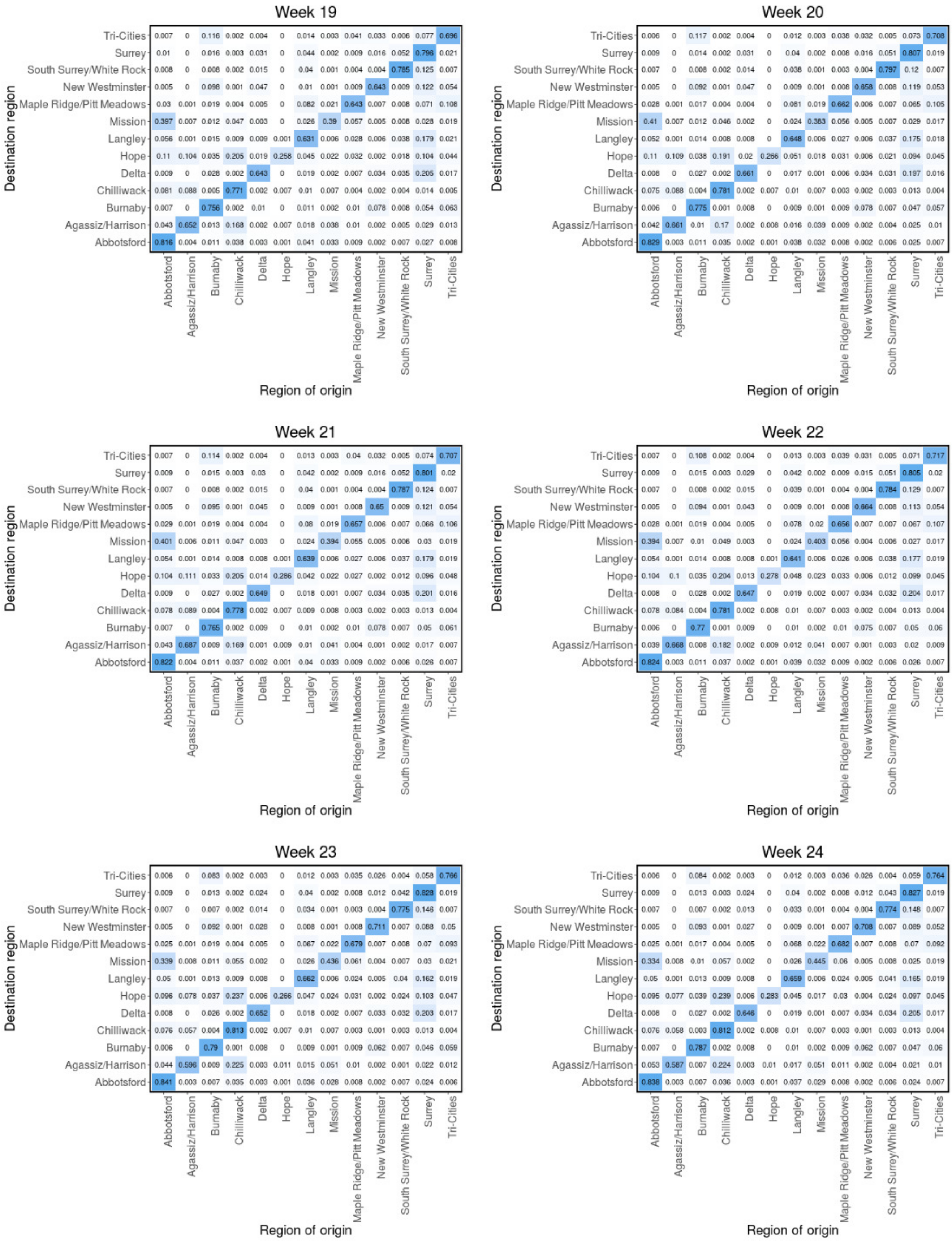

*Figure E: **Mobility matrices**. Probability matrix (π) computed from the Telus mobility data for week 1 - 6 (Nov 5, 2020 - Dec 16, 2020). π_{ji} is the probability that an individual who migrated from one of the 13 LHAs to region j, originated from region i.*

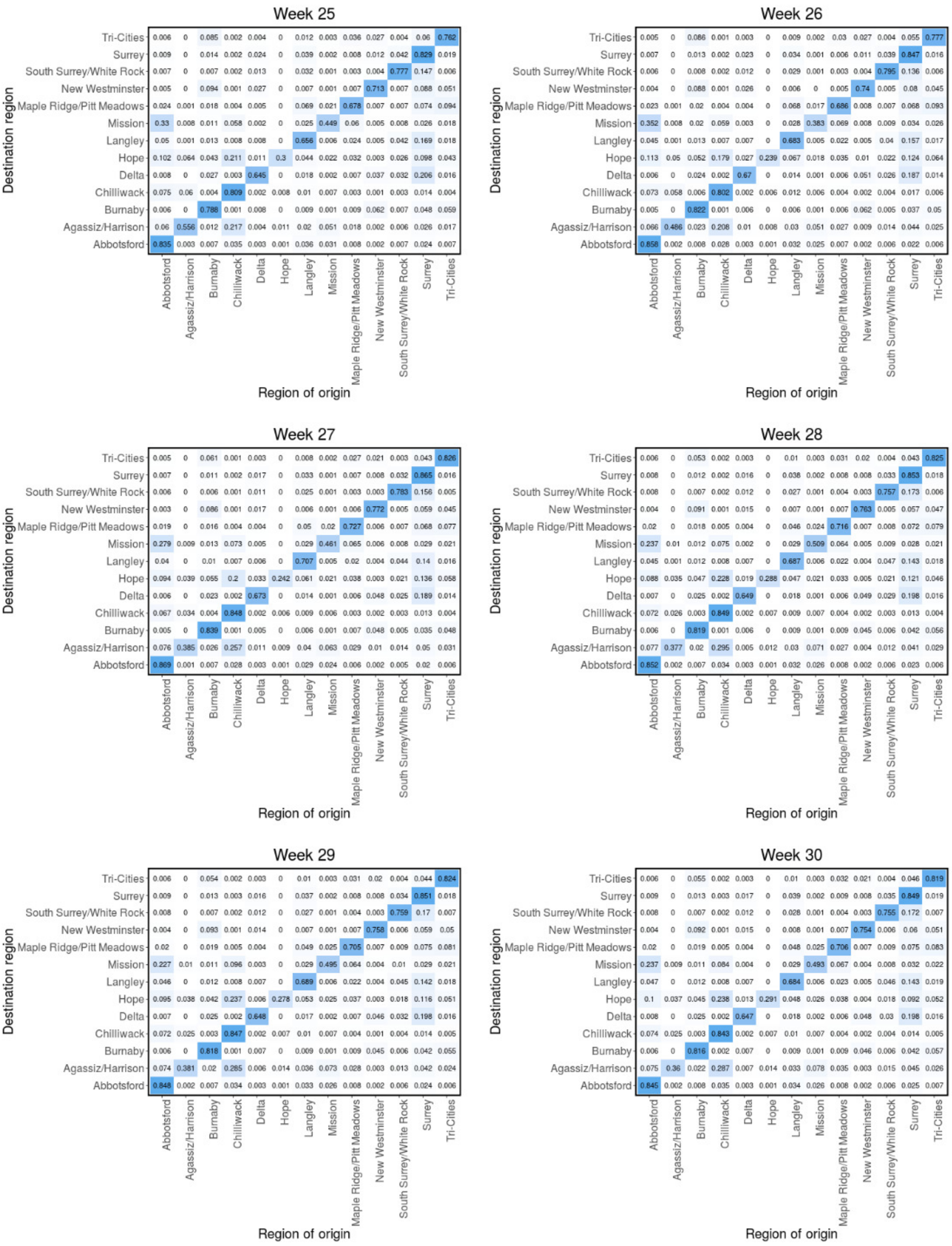

Figure F: ***Mobility matrices****. Probability matrix (π) computed from the Telus mobility data for week 1 - 6 (Dec 17, 2020 - Jan. 27, 2021). π_{ji} is the probability that an individual who migrated from one of the 13 LHAs to region j, originated from region i.*

C Reported cases and model predictions

In this section, we present the model's predicted cases and the estimated parameters for the remaining nine LHA whose results are not presented in the Result section of the main text.

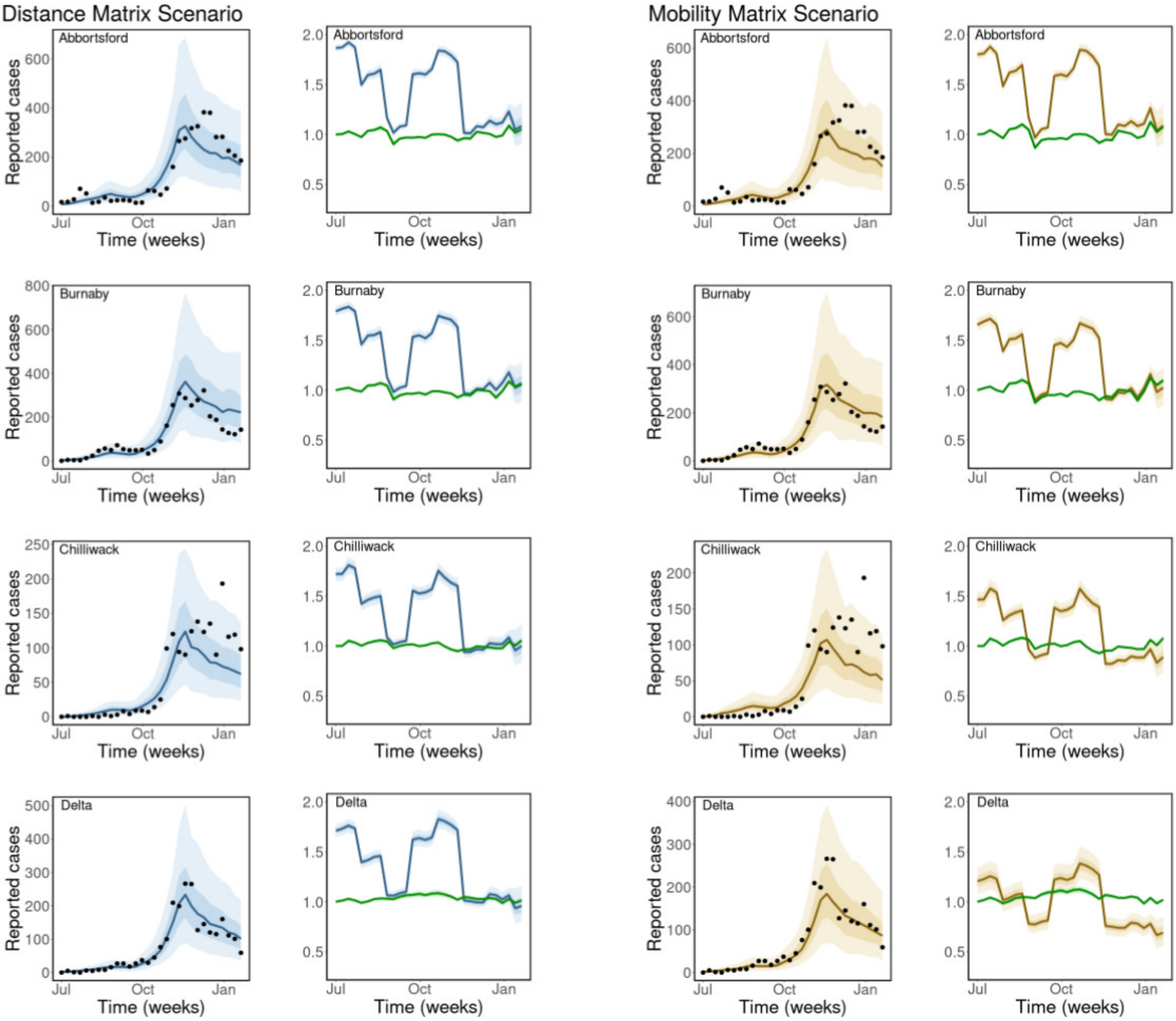

Figure G: ***Observed and estimated COVID-19 cases****. Weekly reported cases and model prediction (columns 1 and 3). Disease transmission rate, $\beta_j(t)$ and the contribution of mobility to disease transmission, $e^{c_1 m_j(t)}$ (columns 2 and 4). Scenarios: fixed distance matrix (blue) and weekly mobility matrices (gold). Black dots are the weekly reported cases of COVID-19, the solid lines are the mean estimates of cases/parameters, the darker bands are the 50% CrI, while the lighter bands are the 90% CrI.*

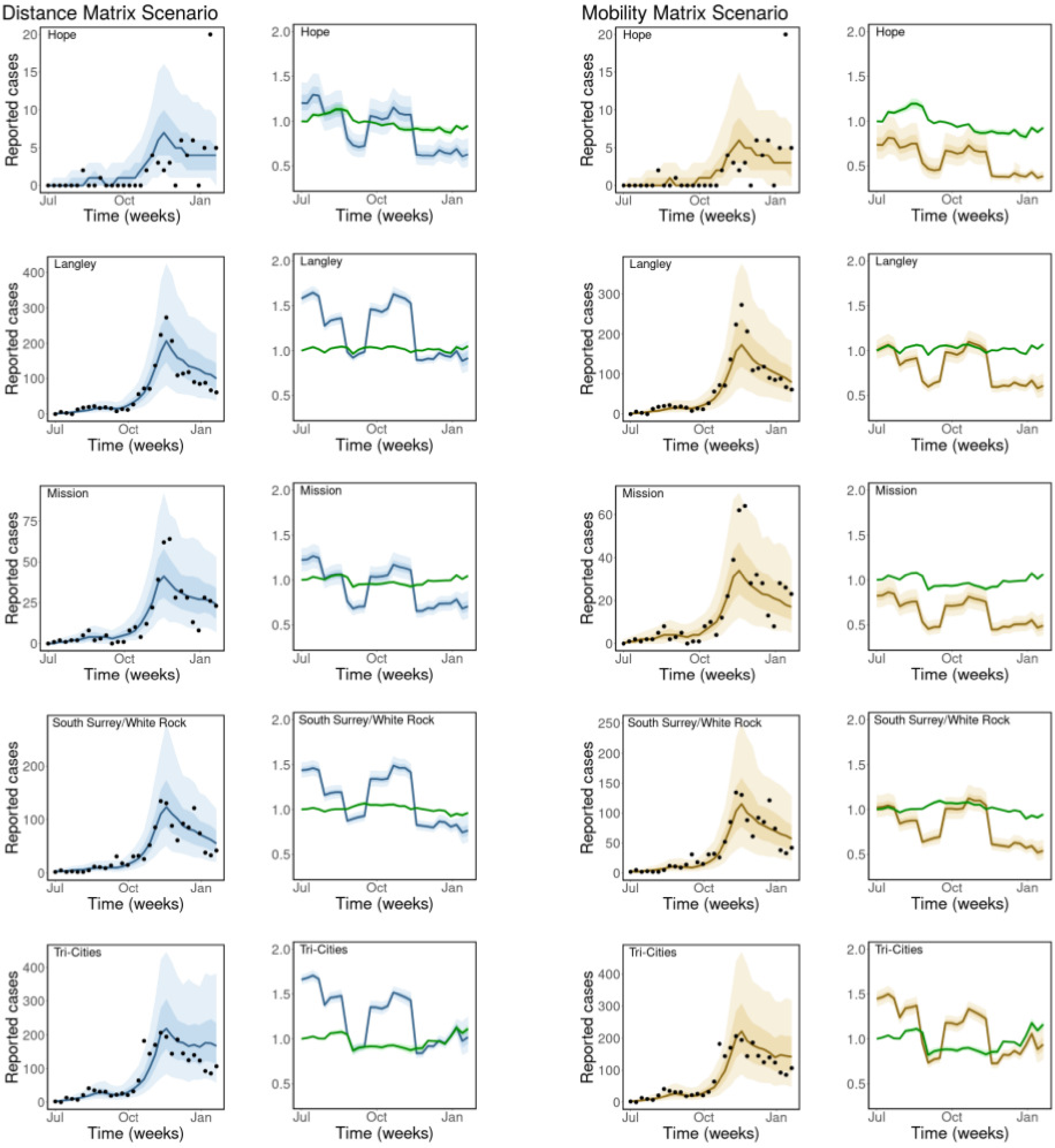

Figure H: ***Observed and estimated COVID-19 cases****. Weekly reported cases and model prediction (columns 1 and 3). Disease transmission rate,* $\beta_j(t)$ *and the contribution of mobility to disease transmission,* $e^{c_1 m_j(t)}$ *(columns 2 and 4). Scenarios: fixed distance matrix (blue) and weekly mobility matrices (gold). Black dots are the weekly reported cases of COVID-19, the solid lines are the mean estimates of cases/parameters, the darker bands are the 50% CrI, while the lighter bands are the 90% CrI.*

.

D Table of estimated parameters

We present the estimated parameters for the distance matrix and weekly mobility matrices scenarios.

Table A: ***Estimated parameters for the distance matrix scenario.*** *The estimated parameters obtained for the distance matrix scenario with* 90% *credible interval (CrI).*

**Parameter**	**Mean estimate (90% CrI)**
i_0	47.61 (44.82, 50.31)
c_{0j} (pop. mean)	0.45 (0.35, 0.54)
c_{0j} (pop. variance)	0.18 (0.12, 0.26)
c_{0_1}	0.62 (0.60, 0.64)
c_{0_2}	0.41 (0.28, 0.56)
c_{0_3}	0.58 (0.55, 0.61)
c_{0_4}	0.54 (0.50, 0.58)
c_{0_5}	0.54 (0.50, 0.57)
c_{0_6}	0.18 (0.00, 0.36)
c_{0_7}	0.46 (0.41, 0.50)
c_{0_8}	0.20 (0.09, 0.30)
c_{0_9}	0.37 (0.31, 0.43)
$c_{0_{10}}$	0.52 (0.45, 0.58)
$c_{0_{11}}$	0.36 (0.31, 0.42)
$c_{0_{12}}$	0.65 (0.646, 0.656)
$c_{0_{13}}$	0.51 (0.48, 0.54)
θ	0.53 (0.44, 0.60)
c_1	1.51 (0.90, 2.10)
g (pop. mean)	-0.33 (-0.52, -0.14)
g (pop. variance)	0.30 (0.16, 0.47)
g_2	-0.19 (-0.21, -0.17)
g_3	-0.50 (-0.52, -0.48)
g_4	-0.12 (-0.14, -0.10)
g_5	-0.02 (-0.03, -0.003)
g_6	-0.57 (-0.60, -0.54)
g_7	-0.50 (-0.55, -0.45)
g_8	-0.59 (-0.77, -0.40)

Table B: ***Estimated parameters for the weekly mobility matrices scenario.*** *The estimated parameters obtained for the scenario we used weekly mobility matrices with* 90% *credible interval (CrI).*

**Parameter**	**Mean estimate** (90% **CrI)**
i_0	50.19 (47.37, 53.04)
c_{0j} (pop. mean)	0.21 (0.05, 0.36)
c_{0j} (pop. variance)	0.35 (0.24, 0.47)
c_{0_1}	0.59 (0.56, 0.61)
c_{0_2}	0.25 (0.06, 0.45)
c_{0_3}	0.50 (0.47, 0.54)
c_{0_4}	0.38 (0.33, 0.43)
c_{0_5}	0.19 (0.08, 0.30)
c_{0_6}	-0.30 (-0.54, -0.04)
c_{0_7}	0.01 (-0.10, 0.11)
c_{0_8}	-0.19 (-0.36, -0.03)
c_{0_9}	-0.02 (-0.15, 0.12)
$c_{0_{10}}$	0.34 (0.21, 0.45)
$c_{0_{11}}$	0.02 (-0.09, 0.15)
$c_{0_{12}}$	0.601 (0.597, 0.61)
$c_{0_{13}}$	0.37 (0.31, 0.43)
θ	0.90 (0.72, 0.98)
c_1	2.11 (1.52, 2.69)
g (pop. mean)	-0.28 (-0.45, -0.10)
g (pop. variance)	0.32 (0.19, 0.50)
g_2	-0.16 (-0.17, -0.14)
g_3	-0.48 (-0.50, -0.45)
g_4	-0.08 (-0.10, -0.06)
g_5	0.02 (0.003, 0.04)
g_6	-0.53 (-0.56, -0.50)
g_7	-0.47 (-0.51, -0.43)
g_8	-0.58 (-0.74, -0.40)

.

E Regions and their population sizes

Table C: ***List of regions and their population sizes.*** *The thirteen local health areas (LHAs) in Fraser Health, British Columbia, Canada, and their population sizes. Source [1].*

	Local Health Authority (LHA)	Population
1	Abbortsford	161, 912
2	Agassiz/Harrison	10, 770
3	Burnaby	257, 926
4	Chilliwack	105, 862
5	Delta	112, 259
6	Hope	8, 931,
7	Langley	161, 725
8	Mission	47, 652
9	Maple Ridge/Pitt Meadows	111, 502
10	New Westminster	82, 590
11	South Surrey/White Rock	107, 347
12	Surrey	512, 436
13	Tri-Cities	254, 583

F Results for the scenario with a fixed mobility matrix for the entire study period

In this section, we consider a scenario where mobility is described by fixed matrix computed from the Telus mobility data for the entire study period.

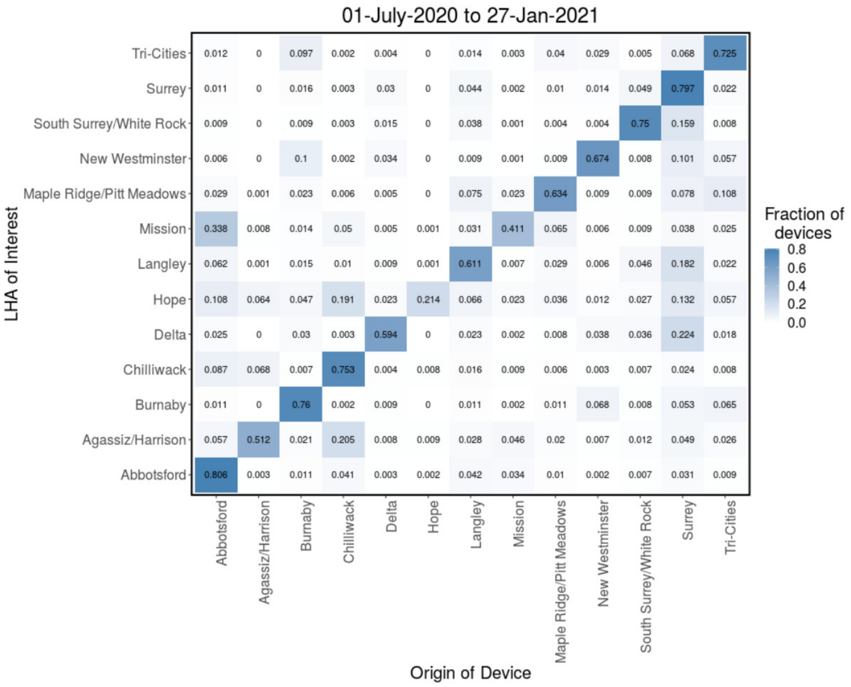

Figure I: ***Fixed mobility matrix for the entire study period.*** *Probability matrix (π) computed from the Telus mobility data for the entire study period from July 1, 2020 to Jan. 27, 2021. π_{ji} is the probability that an individual who migrated from one of the 13 LHAs to region j, originated from region i.*

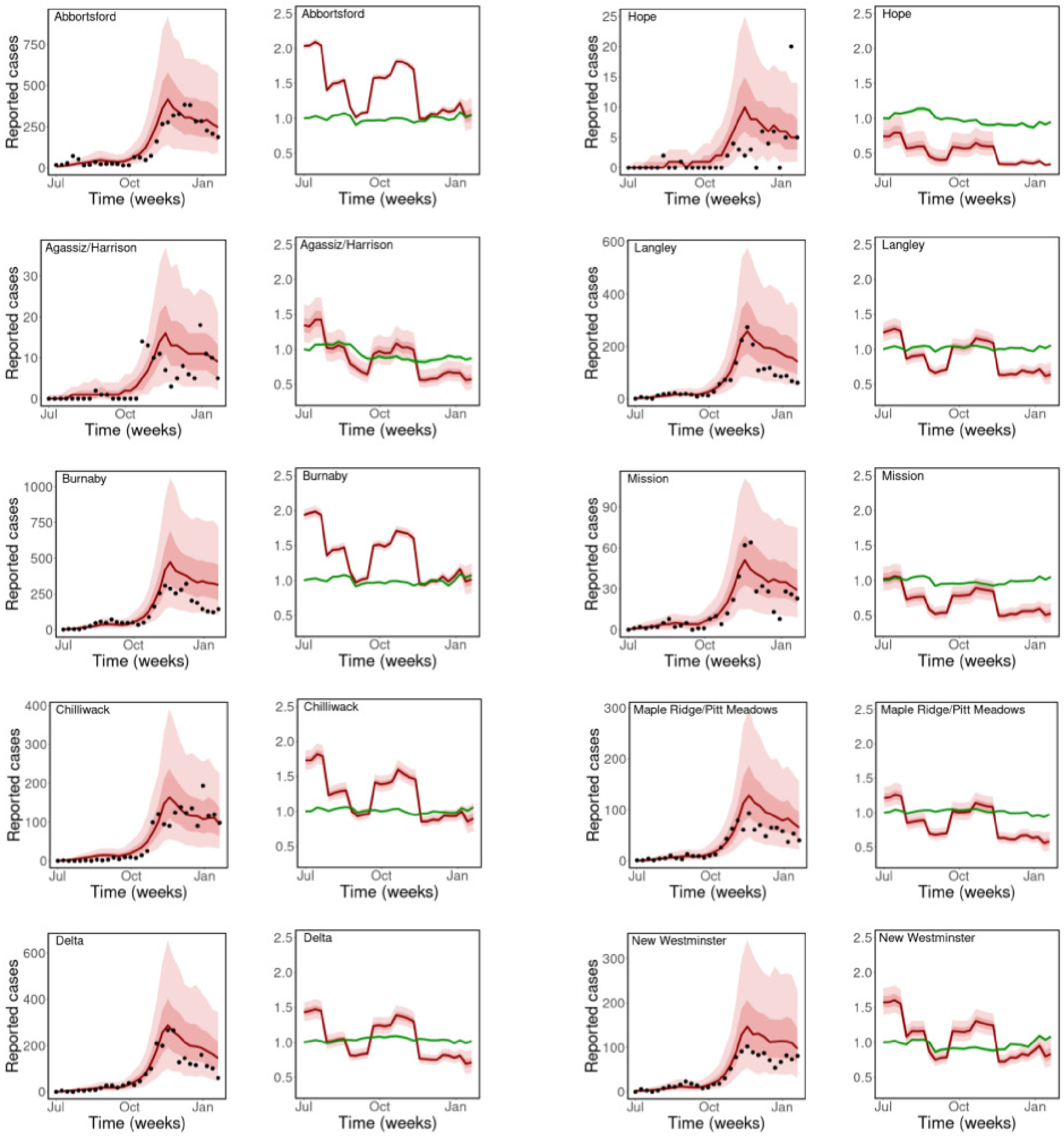

Figure J: ***Observed and estimated COVID-19 cases****. Weekly reported cases and model prediction (columns 1 and 3), computed using the fixed mobility matrix in Figure I. Disease transmission rate, $\beta_j(t)$ and the contribution of mobility to disease transmission, $e^{c_1 m_j(t)}$ (columns 2 and 4). Black dots are the weekly reported cases of COVID-19, the solid lines are the mean estimates of cases/parameters, the darker bands are the 50% CrI, while the lighter bands are the 90% CrI.*

.

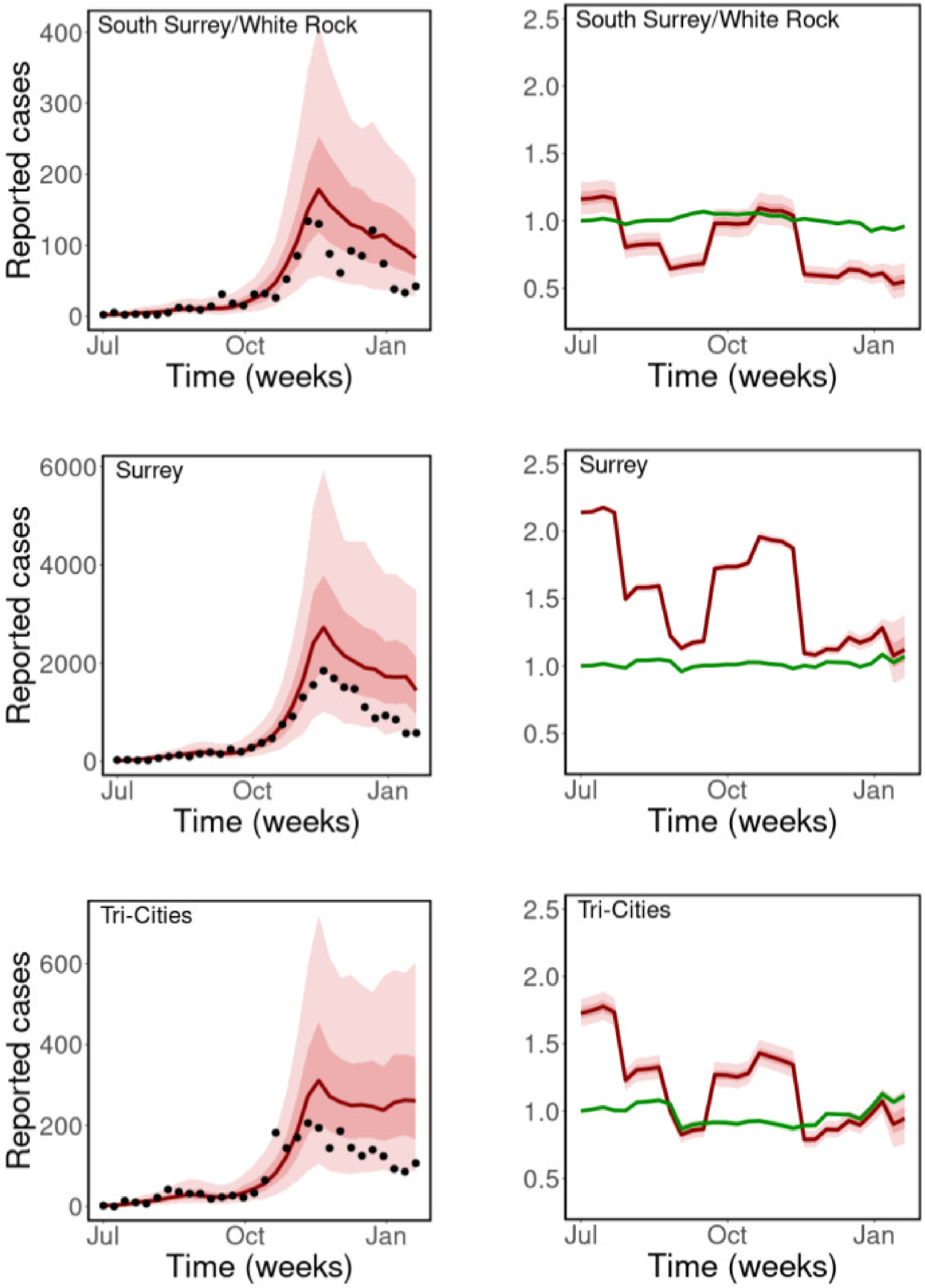

Figure K: ***Observed and estimated COVID-19 cases****. Weekly reported cases and model prediction (columns 1 and 3), computed using the fixed mobility matrix in Figure I. Disease transmission rate, $\beta_j(t)$ and the contribution of mobility to disease transmission, $e^{c_1 m_j(t)}$ (columns 2 and 4). Black dots are the weekly reported cases of COVID-19, the solid lines are the mean estimates of cases/parameters, the darker bands are the 50% CrI, while the lighter bands are the 90% CrI.*

Table D: ***Estimated parameters for the fixed mobility matrix scenario.*** *Estimated parameters with* 90% *credible interval (CrI).*

**Parameter**	**Mean estimate** (90% **CrI)**
i_0	35.75 (33.79, 37.78)
c_{0j} (pop. mean)	0.31 (0.14, 0.48)
c_{0j} (pop. variance)	0.38 (0.26, 0.52)
c_{0_1}	0.71 (0.69, 0.73)
c_{0_2}	0.30 (0.10, 0.50)
c_{0_3}	0.66 (0.62, 0.70)
c_{0_4}	0.54 (0.47, 0.63)
c_{0_5}	0.36 (0.26, 0.45)
c_{0_6}	-0.29 (-0.56, 0.01)
c_{0_7}	0.21 (0.11, 0.32)
c_{0_8}	0.02 (-0.16, 0.18)
c_{0_9}	0.19 (0.07, 0.32)
$c_{0_{10}}$	0.45 (0.34, 0.58)
$c_{0_{11}}$	0.15 (0.05, 0.25)
$c_{0_{12}}$	0.76 (0.756, 0.77)
$c_{0_{13}}$	0.55 (0.49, 0.61)
θ	0.60 (0.52, 0.70)
c_1	1.55 (1.10, 1.99)
g (pop. mean)	-0.41 (-0.60, -0.21)
g (pop. variance)	0.31 (0.17, 0.48)
g_2	-0.34 (-0.36, -0.32)
g_3	-0.60 (-0.62, -0.57)
g_4	-0.22 (-0.24, -0.20)
g_5	-0.11 (-0.13, 0.10)
g_6	-0.67 (-0.71, -0.64)
g_7	-0.60 (-0.64, -0.55)
g_8	-0.71 (-0.92, -0.51)

.

References

[1] BCStats. *Population Estimates & Projections for British Columbia*, (accessed August 11, 2022). https://bcstats.shinyapps.io/popApp/.
